# Lingua Franca of Cardiogenic Shock: Speaking the Same Language

**DOI:** 10.3389/fcvm.2021.691232

**Published:** 2021-09-22

**Authors:** Ashleigh Long, David A. Baran

**Affiliations:** Sentara Advanced Heart Failure Center, Norfolk, VA, United States

**Keywords:** risk score, cardiogenic shock, mechanical circulatory (MCS) support, intraaortic balloon counter pulsation, Impella^®^, classification

## Abstract

Cardiogenic shock has remained a vexing clinical problem over the last 20 years despite progressive development of increasingly capable percutaneous mechanical circulatory support devices. It is increasingly clear that the published trials of various percutaneous mechanical circulatory support devices have compared heterogenous populations of cardiogenic shock patients, and therefore have not yielded a single result where one approach improved survival. To classify patients, various risk scores such as the CARDSHOCK and IABP-Shock-II scores have been developed and validated but they have not been broadly applied. The Society for Cardiac Angiography and Intervention Expert Consensus on Classification of Cardiogenic Shock has been widely studied since its publication in 2019, and is reviewed at length. In particular, there have been numerous validation studies done and these are reviewed. Finally, the directions for future research are reviewed.

## Introduction

Shock is a life threatening condition with circulatory failure leading to inadequate delivery of oxygen to tissues, leading to ischemic dysfunction and injury. This can occur from a variety of causes, including hypovolemia, hemorrhage, or severe infection associated with sepsis. As well, it may occur with pump failure as a primary event. This may occur suddenly such as patients with acute myocardial infarction, or sub acutely such as is seen with acutely decompensated states of chronic heart failure, Cardiogenic shock (CS) is defined as a complex physiological state involving tissue hypoxia and end-organ damage secondary to a failure of the heart to provide adequate systemic perfusion. It remains a significant cause of mortality and morbidity, despite advancing techniques in management (1). The management of cardiogenic shock is complex and beyond the scope of the current work but several recent reviews are available to guide the reader (2–5).

The last significant improvement in survival occurred following the SHOCK trial (more than 20 years ago) and use of immediate revascularization for acute myocardial infarction with CS (6). Compounding this is the lack of consensus regarding degrees of CS severity with related management recommendations. Prior CS trials have enrolled a mixture of patients of various grades of severity. Some, such as patients who are survivors of out of hospital cardiac arrest, may have significant neurologic impairment which determines their outcome, regardless of treatment. Some patients have modest signs of CS vs. others who are on numerous pressors, yet most trials do not distinguish between groups.

## Mortality Risk Prediction Scores

Early assessment of shock severity is critical to identifying patients at the highest risk of mortality and those most likely to benefit from intervention. Previously established cardiogenic shock risk score paradigms include CARD-SHOCK (7), and IABP-SHOCK 2 scores (8). These were derived from prior studies and then subsequently validated. Both demonstrate nearly equivalent predictive ability for intra-hospital, short term mortality, even when accounting for operator experience (9). For both scores however, comparative assessments have shown that predictive accuracy is acceptable with CS secondary to Acute Coronary Syndrome (ACS), but not other causes (9, 10). Additionally, neither score was designed to accommodate serial assessments, or deteriorating clinical status.

## Use of Support Devices

Currently there is little data to guide the evidence-based use of mechanical support devices in cardiogenic shock, though multiple trials have been performed in the last two decades. One of the largest trials was the multicenter, randomized trial, the IABP-SHOCK 2 study (11, 12). This trial studied utility of mechanical hemodynamic support with Intra-aortic balloon pump counter pulsation (IABP), a circulatory support device which increases myocardial perfusion directly, and indirectly increases cardiac output through afterload reduction. Here, IABP use for hemodynamic support vs. control was assessed in patients with ACS and CS undergoing revascularization. IABP was typically placed following revascularization. With a sample of size of 600 patients followed to 6 years post study enrollment, there was no difference in mortality rate noted between those receiving IABP and those in the control arm of the study at any time point assessed (12).

The IMPRESS CP trial was a randomized comparison of IABP vs. Impella CP in patients with AMI-CS and receiving mechanical ventilation (13). The timing of device placement was left to the discretion of the operator, with more than 80% of patients having a support device placed after PCI. Interestingly, there was no difference in mortality noted in either arm at 30 days or 6 months. In both trial arms reduced mortality was noted when mechanical support devices were placed early, typically prior to PCI. This was a surprising result since the patients were critically ill, all receiving mechanical ventilation and unable to consent. Furthermore, the Impella CP device clearly provides much more cardiac flow than an IABP. However, the trial didn't measure shock severity or resolution of shock but mortality which can be greatly influenced by neurological status. Whether any device would successfully salvage such patients remains an open question.

Noting the lack of a “lingua franca” for CS, the Society for Cardiac Angiography and Intervention convened an expert group and released a proposed classification in 2019 (14). This was the result of a multi-disciplinary writing group and sought to provide a common framework for use by clinicians and researchers alike. This classification scheme emphasizes ease of use across the spectrum of care, from pre-hospital to intensive care and catheterization laboratory and the facilitation of communication between all members of the treatment team. It was hoped that this framework would create a standardized platform to be used for clinical trials and research going forward.

**Figure 1:**
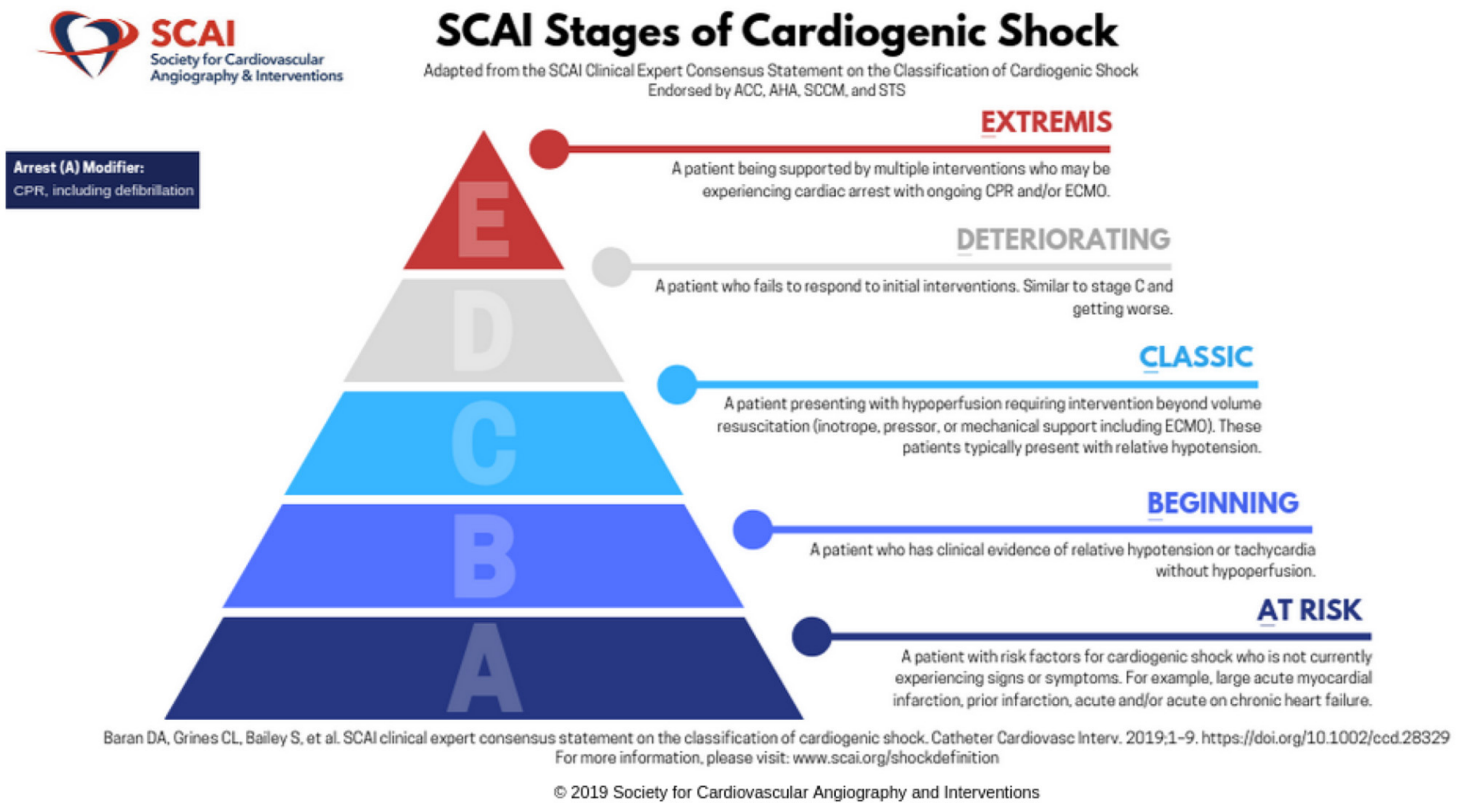
The SCAI Shock classification system. *Reprinted by permission from Society for Cardiovascular Angiography and Interventions*. © *2021 Society for Cardiovascular Angiography and Interventions (SCAI). All rights reserved*.

This expert consensus document was endorsed by the American College of Cardiology, the American Heart Association, the Society of Critical Care Medicine, and the Society of Thoracic Surgeons (14). As shown in Figure 1, there are five stages “A–E,” with each increasing stage indicative of deterioration in the patient's clinical and hemodynamic status. Stage A is “at risk” for CS, stage B is “beginning” shock, stage C is “classic” CS, stage D is “deteriorating”, and E is “extremis”. The criteria are also meant to alert providers regarding changes in the patient's clinical status. The staging system was developed without any preceding evidence that it would accurately predict outcomes or prove to be valid. Given the broad multidisciplinary representation, the goal of implementing a widespread validation and use of the staging system seemed reasonable, and the hope was that it might lead to improvements in design of future trials.

## Validation of Scai Shock Classification

There have been several large retrospective analyses (15–23) and one prospective study (24) since the SCAI shock stages were published in 2019. This framework has been shown to predict mortality when applied across multiple categories and in different scenarios. These included SCAI classifications made at time of initial triage or during inpatient ICU admissions (16, 18, 19), and those with out of hospital cardiac arrest (17, 21). The SCAI shock stage was also associated with prognosis in patients with acute coronary syndrome or decompensated heart failure (15, 22, 23).

Additionally, the first prospective validation of the SCAI shock criteria was recently published, which demonstrated that initial SCAI Stage was a strong predictor of survival, with thirty-day survival strongly correlated with initial SCAI shock stage 100, 65.4, 44.2, and 60% for patients with initial SCAI shock stage B, C, D, and E respectively (*p* = 0.0004) (24). Age and initial SCAI Shock Stage were shown to be the strongest predictors of survival by Cox proportional hazards. In addition, the group showed that 24-h re-assessment was critically important. If a patient improved in SCAI stage (lower degree of CS), then the mortality was significantly lower. Conversely, if SCAI stage is not changed or worsens at 24 h, the survival is much worse. These findings have great practical importance. If a patient is in a hospital without access to the full breadth of support strategies but is improving with management of cardiogenic shock, the outlook is positive. However, if the patient is not improving at 24 h, it serves as a strong indicator to alter the course of care, if appropriate as the predicted outcome is not good.

## Implementation Of Staging System By All Care Team Members

The simplicity of the SCAI criteria facilitates its use by any member of the patient's care team, from initial assessment and triage by Shock team responders and emergency department staff, to extracting objective data from the patient's electronic medical record (EMR) after admission. In fact, the SCAI classification system could potentially be integrated into an EMR to facilitate awareness of a patient's clinical status for the entire care team, and to alert providers of deteriorating clinical status, which may require associated escalating interventions. This wouldn't be perfect, but could be based on vital signs, changes in laboratories and urine output, since most of these factors are integrated into the system already.

## Future Directions

The SCAI Shock classification has been validated in retrospective as well as prospective cohorts and has gained traction since it filled a void which had existed. Future directions include refinements to the classification to guide clinicians and increase uniformity of assignment of SCAI stage. The writing committee which created the SCAI Shock Staging is currently working on an updated guidance document which will offer more concrete definitions of the various SCAI stages, while maintaining the simplicity and utility which the system enjoys currently. It is notable that the SCAI staging has been found to be predictive with a variety of populations and ways of retrospectively and prospectively defining it. The key elements appear to be hypoperfusion at the gateway to SCAI Stage C, and the element of time indicating that a patient is deteriorating (stage D). The specific laboratory or hemodynamic values seem less important than the clinical gestalt, as shown by the prospective experience.

Studying CS patients in the setting of prospective randomized trials is challenging for a number of reasons. First, these patients have poor perfusion by definition so obtaining informed consent is problematic. Given the patient acuity, it is often not practical to wait for prolonged periods of time to find designated family representatives and surrogate consent is not always acceptable. In addition, depending on the study entry criteria, patients may be excluded due to lack of a catheter or other datapoint despite the presence of CS. Furthermore, despite few proven therapies, there is a frequent lack of equipoise. Investigators often believe that mechanical pumps must be better given the increase in cardiac output, and may be unwilling to randomize some patients. Lastly, prior to the SCAI Shock classification, all patients with CS were “lumped together” leading to a mix of outcomes.

Another way to study CS is through registry studies. The American Heart Association is exploring the possibility of a large nationwide registry of CS patients which would gather broad data across a variety of centers of varying size and experience across the United States and give unique insights. The focused Cardiogenic Shock Working Group has utilized their registry to generate insights into CS outcomes (15, 23).

Perhaps the most impactful change would be the use of the SCAI stages as part of prospective, randomized clinical trials of treatments for CS. As stated earlier, trials which include extremely heterogeneous populations have not shown superiority of any device to modify survival in patients with cardiogenic shock. Either that means that cardiogenic shock is not a modifiable condition (which is unlikely), or that the different subgroups of patients behave differently. Designing future clinical trials and large prospective studies where the SCAI classification system is used to define patient responder subgroups is a tangible goal for the imminent future. Conceivably this would lead to further refinements of the SCAI criteria, with specific algorithms for management by patient responder group, and new systems for cardiogenic shock management.

## Conclusion

A small fire is easier to quell than a massive blaze, and treating all shock in a similar fashion is like using a fire extinguisher to put out all fires: Doomed to failure! Hopefully, the lingua franca of shock (the SCAI Shock classification) will lead to a new chapter being written where we find effective treatments to reduce the mortality of this devastating illness. After more than 20 years of trying, we owe our patients nothing less than persistence and to find treatments that work.

## Data Availability Statement

The original contributions presented in the study are included in the article/supplementary material, further inquiries can be directed to the corresponding author.

## Author Contributions

DB conceived of the paper and edited and revised manuscript. AL wrote the first draft and edited for clarity and content. Both authors contributed to the article and approved the submitted version.

## Conflict of Interest

DB has consulted with Abiomed, Abbott, Getinge, Livanova and is on steering committee of Procyrion and CareDx. He has spoken for Pfizer. None of these are related to the current work. The remaining author declares that the research was conducted in the absence of any commercial or financial relationships that could be construed as a potential conflict of interest.

## Publisher's Note

All claims expressed in this article are solely those of the authors and do not necessarily represent those of their affiliated organizations, or those of the publisher, the editors and the reviewers. Any product that may be evaluated in this article, or claim that may be made by its manufacturer, is not guaranteed or endorsed by the publisher.
